# TXNDC5, a Newly Discovered Disulfide Isomerase with a Key Role in Cell Physiology and Pathology

**DOI:** 10.3390/ijms151223501

**Published:** 2014-12-17

**Authors:** Elena Horna-Terrón, Alberto Pradilla-Dieste, Cristina Sánchez-de-Diego, Jesús Osada

**Affiliations:** 1 Grado de Biotecnología, Universidad de Zaragoza, Zaragoza E-50013, Spain; E-Mails: elenahornaterron@gmail.com (E.H.-T.); albertopradilladieste@gmail.com (A.P.-D.); csanchezdg@gmail.com (C.S.-D.); 2 Departamento Bioquímica y Biología Molecular y Celular, Facultad de Veterinaria, Instituto de Investigación Sanitaria de Aragón (IIS), Universidad de Zaragoza, Zaragoza E-50013, Spain; 3 CIBER de Fisiopatología de la Obesidad y Nutrición, Instituto de Salud Carlos III, Madrid E-28029, Spain

**Keywords:** thioredoxin domain containing 5 (TXNDC5), protein disulfide isomerase (PDI), endoplasmic reticulum 46 (Erp46), PDI15, thioredoxin-related protein in the cell plasma (PC-TRP), endo PDI

## Abstract

Thioredoxin domain-containing 5 (TXNDC5) is a member of the protein disulfide isomerase family, acting as a chaperone of endoplasmic reticulum under not fully characterized conditions As a result, TXNDC5 interacts with many cell proteins, contributing to their proper folding and correct formation of disulfide bonds through its thioredoxin domains. Moreover, it can also work as an electron transfer reaction, recovering the functional isoform of other protein disulfide isomerases, replacing reduced glutathione in its role. Finally, it also acts as a cellular adapter, interacting with the *N*-terminal domain of adiponectin receptor. As can be inferred from all these functions, TXNDC5 plays an important role in cell physiology; therefore, dysregulation of its expression is associated with oxidative stress, cell ageing and a large range of pathologies such as arthritis, cancer, diabetes, neurodegenerative diseases, vitiligo and virus infections. Its implication in all these important diseases has made TXNDC5 a susceptible biomarker or even a potential pharmacological target.

## 1. Introduction

The thioredoxin domain containing protein 5 (TXNDC5), also known as resident endoplasmic reticulum 46 (Erp46); protein disulfide isomerase family A, member 15 (PDI15); thioredoxin-related protein in the cell plasma (PC-TRP) or endo PDI; is a protein-disulfide isomerase. In fact, it is claimed to have thioredoxin (TRX) (Trx-like domains) that catalyze its thioredoxin activity and enable it to act as a chaperone in the endoplasmic reticulum (ER) [1]. There is evidence to suggest that TXNDC5 expression is induced by hypoxia in endothelial cells [2] and endothelium of tumors [2]. TXNDC5 is upregulated in several cancers such as hepatocellular [3], breast, cervical, colon, esophageal, liver, lung, stomach and uterine carcinomas [4,5], in colorectal cancer [6], in non-small cell lung carcinoma [7] and renal tumor tissue [8]. Due to the anaerobic metabolism of cancer cells [4], it has been stated that their proliferation could be due to hypoxia and this would induce TXNDC5. This notion has been supported by studies in various diseases in which oxygen is limited, such as diabetes [9], arthritis [10,11,12], neurodegenerative diseases [13] and vitiligo [14]. However, hypoxia does not always induce TXNDC5 upregulation. For example, in non-small cell lung carcinoma, hypoxia does not change TXNDC5 expression [7], leaving an open horizon to better characterize its regulation. This wide spectrum of changes and controversies warrants further research in the future.

**Figure 1:**
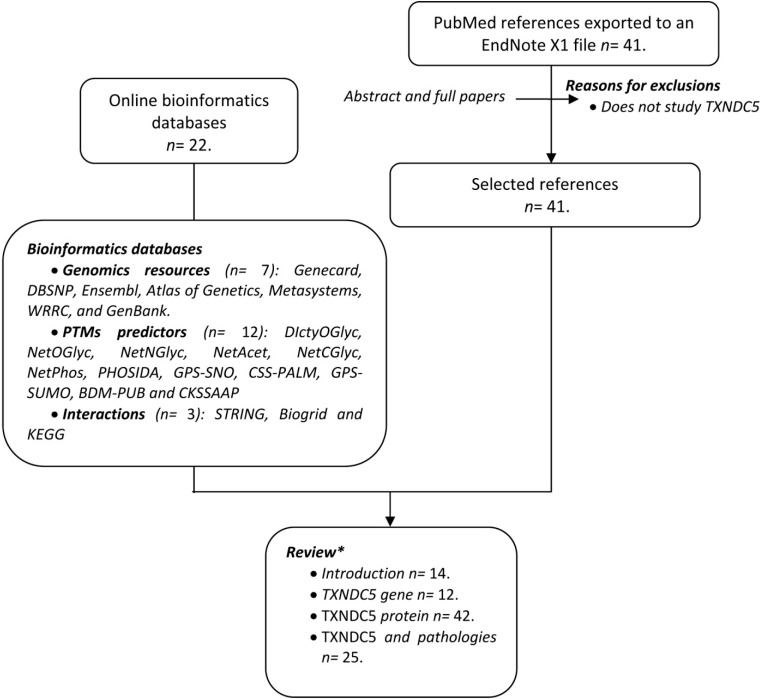
Flow chart displaying the information collection process. Two different sources of data were used: Data from online bioinformatics databases and a search in PubMed. EndNote X1 (Thomson Reuters, New York, NY, USA). * Some references may appear in more than one section of the review.

Since TXNDC5 was discovered in 2003, a large amount of miscellaneous information has been published. To make advances in the field, the present report has adhered to systematic review guidelines [15]. All the data has been collected according to the criteria shown in Figure 1. The search in PubMed (http://www.ncbi.nlm.nih.gov/pubmed/) using the keywords (TXNDC5 or Erp46) identified 40 hits from November 1945 to July 2014. In addition, a search in electronic databases was carried out. The combined information of the two sources has been the basis of this review.

## 2. *TXNDC5* Gene

*TXNDC5* is a gene placed in the negative chain of *Homo sapiens* chromosome 6, particularly in the 6p24.3 position, with a length of 29,565 bp. This gene contains 10 exons and 9 introns [1].

### 2.1. Orthologous Genes

The *TXNDC5* gene has also been found in other species, with orthologous genes in chimpanzee, mouse, rat, cow and dog, among others. Table 1 shows the orthologous genes most similar to genes found in *Homo sapiens*, accompanied by their taxonomic classification, and their names [1].

### 2.2. Paralogous Genes

Groups of genes in different chromosomal locations in the same organism are paralogous. Their structure is similar, since they have evolved from the same ancestral gene. There are different *TXNDC5* paralogs in humans, such as *DNAJC10*, *PDIA6* or *PDIA5*. The first gene encodes for an endoplasmic reticulum (ER), chaperone, a part of a complex involved in recognition and degradation of misfolded proteins. This co-chaperone reduces incorrect disulfide bonds in misfolded glycoproteins. For its part, the *PDIA6* gene encodes for a disulfide isomerase in the ER, responsible for formation, reduction and isomerization of disulfide bonds in proteins. Therefore it is thought to play a role in the folding of proteins with disulfide bonds. The last one, *PDIA5*, encodes member 5 of the protein disulfide isomerase family [1].

**Table 1:** Orthologs of the human *TXNDC5* gene.

Organism	Taxonomic Classification	Gene	Human Similarity
Chimpanzee (*Pan troglodytes*)	Mammalia	*TXNDC5*	99.44 (*n*) 98.98 (*a*)
Mouse (*Mus musculus*)	Mammalia	*TXNDC5*	86.81 (*n*) 89.57 (*a*)
Rat (*Rattus norvegicus*)	Mammalia	*TXNDC5*	85.83 (*n*) 89.84 (*a*)
Cow (*Bos taurus*)	Mammalia	*TXNDC5*	85.42 (*n*) 89.33 (*a*)
Dog (*Canis familiaris*)	Mammalia	*TXNDC5*	85.91 (*n*) 87.76 (*a*)
Opossum (*Monodelphis domestica*)	Mammalia	*TXNDC5*	78 (*a*)
Platypus (*Ornithorhynchus anatinus*)	Mammalia	*TXNDC5*	69 (*a*)
Chicken (*Gallus gallus*)	Aves	*TXNDC5*	74.53 (*n*) 74.53 (*a*)
Lizard (*Anolis carolinensis*)	Reptilia	*TXNDC5*	66 (*a*)
African clawed frog (*Xenopus laevis*)	Amphibia	BC045245.1	76.01 (*n*)
Tropical clawed frog (*Xenopus tropicalis*)	Amphibia	MGC75894	76.45 (*n*)
Zebrafish (*Danio rerio*)	Actinopterygii	Dr.25420	76.05 (*n*)
Rainbow trout (*Oncorhynchus mykiss*)	Actinopterygii	BX307368.1	75.21 (*n*)
Sea squirt (*Ciona intestinalis*)	Ascidiacea	Cin.2468	73.28 (*n*)
Sea squirt (*Ciona savignyi*)	Ascidiacea	−	44 (*a*)
Fruit fly (*Drosophila melanogaster*)	Insecta	*Prtp*	54.83 (*n*) 47.16 (*a*)
Mosquito (*Anopheles gambiae*)	Insecta	AgaP_AGAP000044	53.96 (*n*) 43.51 (*a*)

The percentage of similarity is shown followed by either (*n*) when the comparison was established using nucleic acid or (*a*) amino acid sequences, respectively.

### 2.3. Gene Polymorphisms

Single nucleotide polymorphisms (SNPs) represent the most common genetic variations in the human genome [16]. Supplementary Table S1 shows a large number of SNPs in the *TXNDC5* gene. As can be seen, all SNPs associated with pathology are placed in non-coding regions of DNA (introns, 5'-UTR and 3'-UTR), a fact that reveals the importance of these regions in gene expression and regulation of *TXNDC5*.

### 2.4. TXNDC5 Transcripts

The *TXNDC5* gene encodes for six transcripts; while all are generated by alternative splicing, only two of them code for a protein (*TXNDC5-001* and *TXNDC5-003*) [17]. *TXNDC5-001* has 2964 bp distributed in 10 exons. It generates isoform 1 of TXNDC5, recognized as a canonical sequence, while *TXNDC5*-*003* has 1301 bp distributed in 8 exons and generates a protein of 389 amino acids (isoform 2), which shares 75% identity with isoform 1; the two differ in the first 108 amino acids [17].

### 2.5. TXNDC5 Transcriptional Regulation

As we mentioned above, the *TXNDC5* gene has two transcriptional start sites, each one coding for one of the functional protein isoforms. Using the DECODE database, which has information based on experimental observations, ten transcription factors that could modulate *TXNDC5* expression were predicted and are compiled in Table 2. The majority of them participate in general cell processes, modulating a wide range of the steps involved in gene transcription, and have ubiquitous expression [17,18]. Most of them are involved in misfolding protein processes, endoplasmic reticulum (ER) stress, hypoxia, differentiation, proliferation, apoptosis and cancer development [18].

**Table 2:** Transcription factors found to control *TXNDC5* gene expression [19,20].

Transcription Factor	Biology Process	Tissue Expression
HTF or HER-2	Growth factor receptor	Epithelial tissue
ATF6	Leucine zipper in response to misfolded proteins via cAMP	Ubiquitous expression
XBP1	Plasma cell differentiation in response to ER stress	Ubiquitous expression
Pax6	Brain and eye morphogenesis	Developing sensory organs, central nervous system and endocrine system
ATF	Differentiation, proliferation and apoptosis	Ubiquitous expression
cMyb	Pro-oncogene	Ubiquitous expression
Max1	Proliferation and apoptosis through H3 Lys-9 methyl-transferase complex	Ubiquitous expression
Arnt	PAH pro-carcinogen activator in response to hypoxia	Ubiquitous expression
USF1	Cell differentiation and proliferation	Ubiquitous expression
	FCHL (Familiar combined hyperlipidemia) transcription factor	Muscle and fat tissue

### 2.6. TXNDC5 Post-Transcriptional Regulation

*TXNDC5* mRNA expression is also regulated by the conjoined gene (CG) *MUTED-TXNDC5* [21]. *MUTED* is a gene related to lysosomes, melanosomes and high-density organelles biosynthesized in platelets. *MUTED-TXNDC5* complex does not have any open reading frame for the gene MUTED sand only the 3'-UTR of the parental gene *TXNDC5* is translated [21]. The presence of the CG *MUTED-TXNDC5* may explain the lack of correlation between mRNA and protein levels in some tissues.

## 3. TXNDC5 Protein

TXNDC5 is a member of the disulfide isomerase (PDI) family [22], mainly expressed in liver and endothelial cells (where it is upregulated in hypoxia) [1,23]. Moreover, it has also been found in brain, spleen, lung, kidney, testis, and pancreas (particularly in β-cells), but it has not been detected in skeletal and heart muscle, or in the peripheral nervous system [1].

Regarding its cellular location, TXNDC5 seems to be mainly expressed in the ER [2]; nevertheless, it is also located in lysosomes, vacuoles, cytosol, Golgi, mitochondrion, plasma membrane and can even be exported to the extracellular medium [1].

### 3.1. Protein Structure

**Figure 2:**
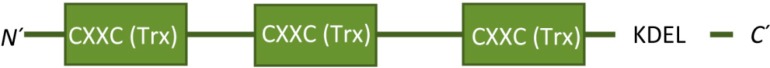
Scheme showing *TXNDC5* domains. The graphic shows the three Trx-like domains, placed between amino acids 14–95, 118–221 and 251–355 [26].

TXNDC5 has three TRX (Trx-like domains), domains which contain a CXXC (two cysteines separated by two other amino acid residues) catalytic sequence, and, therefore, are believed to exert redox activity [24]. As represented in Figure 2, this protein also possesses a hydrophobic *N*-terminal sequence and a *C*-terminal KDEL sequence that retains it in the ER. Furthermore, TXNDC5 has a second hydrophobic helix that can act as an additional transmembrane sequence or as a translocation signal [24]. A third feature of its structure comes from five central β-strands flanked by four α-helices. Conformational changes in this structure may play an important role in the protein function [25]. As a non-globular protein, it can be found as a monomer and dimer in solution, with a molecular mass of 45 and 73 kDa, respectively [25].

### 3.2. Post-Translational Modifications (PTMs)

As a protein, TXNDC5 is susceptible to posttranslational modifications. As shown in Table 3, different web servers can predict where the PTMs can be placed most probably. With the exception of mannosylation, all other potential modifications are plausible.

**Table 3:** Posttranslational modifications of TXNDC5 and participant amino acids.

Modification	Modified Amino Acids	Prediction Server	References
Glycosylation	Thr (174, 304, 306), Ser (308)	DictyOGlyc	[27]
	Thr (174, 299, 302, 304, 306), Ser (183, 308)	NetOGlyc	[28]
	No reports (*)	NetNGlyc	[29]
Acetylation	No reports (**)	NetAcet	[30]
Mannosylation	No reports (***)	NetCGlyc	[31]
Phosphorylation	Thr (138, 167, 174, 306, 335), Ser (62, 108, 125, 183, 285, 364, 392, 409, 412, 419), Tyr (106, 151,192, 289)	NetPhos	[32]
	Thr (174, 304, 306), Ser (62, 108, 125, 129, 183, 197, 238, 292, 308, 392, 409, 412, 419)	Phosida	[33,34]
*S*-nitrosylation	Cys (89, 92, 217, 220, 350, 353)	GPS-SNO	[35]
Palmitoylation	Cys (220)	CSS-Palm	[36]
Sumoylation	Lys (150, 241, 429)	GPS-SUMO	[37,38]
Ubiquitination	Lys (33,68,71,78,116,169,206,208,149,303, 318,334,335, 337), Lys (41,68,71,78)	BDM-PUB	[39]
		CKSSAAP	[40,41]

* NetNGlyc reports no results, so there are no probable glycosylation sites in the *N*-terminal region of TXNDC5. Neither DictyOGlyc nor NetOGlyc report any glycosylation site in the *N*-terminal region; ** NetAct found no Ala, Gly, Ser or Thr to be susceptible to acetylation; *** No results were found using NetCGlyc. Mannosylation is uncommon in mammal proteins, and mainly affects SNC (notwithstanding brain) and skeletal muscle, tissues in which TXNDC5 is not synthesized. All predictions were made using bio-informatics tools that checked homology of TXNDC5 protein domains with experimental databases for the PTMs. The sequence used to predict the PTMs of TXNDC5 was the canonical sequence of isoform 1.

### 3.3. Protein Functions

TXNDC5 catalyzes the rearrangement of disulfide bonds, due to its disulfide isomerase activity. First of all, it reduces the incorrect disulfide bonds formed in the newly folded proteins, and then catalyzes the oxidation of residues to arrange disulfide bonds in the native structure. This process is favored by the redox conditions of the ER [23,42]. For another thing, TXNDC5 has a chaperone activity, independent of its isomerase activity and in which TRX catalytic domains are not involved. It contributes to the oxidative folding of newly synthesized membrane proteins in the ER. Misfolded proteins are recognized by the quality control system of the ER and are marked for degradation via the proteasome [1]. TXNDC5 can also promote the correct protein folding by taking part in electron transfer to other oxidoreductases during the oxidation by Ero1-α-PDI, recovering the semi-reduced or reduced PDI isoform, taking over the role of GSH. However, Ero1-α showed lower affinity for TXNDC5 than other thioreductases (such as Erp44, Erp57 or Erp52) and TXNDC5 has higher oxygen consumption rates than the others [43,44]. On the other hand, the PRX4 protein can oxide a large range of PDIs, having special affinity for TXNDC5, binding the motif TRX, while Ero1α recognizes this protein by contact between a β-hairpin of Ero1α and the hydrophobic pocket of TXNDC5; thus, Ero1α has a lower efficiency than PRX4. As for peroxiredoxin 2, mutation analyses in TXNDC5 have shown that the complex stability of the latter involves disulfide bonds; so reducing its intramolecular disulfide bonds destabilizes the complex. In this way, protein interaction depends on the cell oxidation state. Other results of the differences in the binding mechanisms are the differing specificities of Ero1α and PRX4 toward PDIs, which allows each PDI to play a distinct and significant role during oxidative folding [45,46]. Several studies have shown that the conformation of the peptide rather than its sequence is important in the recognition and binding by TXNDC5. However, sequences usually show a recurrent motif of six residues that is constituted by one or more Phe or Tyr and other large hydrophobic amino acids, which can be interrupted by Lys, Arg or His. It is frequent to find Ser, Gly, Pro or Ala in the *N*-terminal domain [25].

Oxidative stress can disrupt the ER, which causes an abnormal protein folding, and cell apoptosis. As mentioned, TXNDC5 is involved in helping proteins to fold themselves correctly, and it protects cells from entering in apoptotic pathways. In this context, TXNDC5 seems to interact with isoform 2 of NADPH oxidase (Nox2) and this complex inhibits Nox2 activity. TXNDC5 also reduces Ras^C118^, which limits Ras activation and plays an important role in NADPH oxidases and NOX function, modulating Ras expression in an indirect manner. These findings can be related to the antioxidative properties of statins. These agents induce proteins with antioxidative functions (TXNDC5), and downregulate oxidative proteins (Nox2). Thus, these findings give further support to the antioxidative function of TXNDC5 [47,48].

Finally, TXNDC5 has been proposed to act as a cellular adapter, interacting with the *N*-terminal domain of adiponectin receptor 1 (Adipo-R1); thus, enhancing TXNDC5 expression increases the interaction with Adipo-R1 and, indirectly, with AdipoR2 [49,50], an interaction observed in Chinese hamster ovary cells, but not confirmed in renal cell carcinoma [8]. Although immunohistochemistry showed co-localization of Adipo-R1 and ERp46 in membranes of the latter tissue, co-immunoprecipitation studies and crosslinking experiments did not show interactions between these two proteins and a bacterial adenylate cyclase-based two-hybrid assay using *N*-termini of Adipo-R1 and ERp46 revealed no evidence of protein interactions either. However, other transient interactions between Adipo-R1 and ERp46 cannot be ruled out [8]. Likewise, experiments did not evaluate additional proteins or cell conditions, such as oxidative and nitrosative stress, or hyperoxidation and *S*-nitrosylation of ERp46, which might modulate interactions of ERp46, and could affect the interaction between ERp46 and Adipo-R1. Finally, evidence suggests that ERp46’s *N*-terminus might not be the interacting site for Adipo-R1, and that catalytic thioredoxin domains of ERp46 constitute a potential peptide binding site for interactions [25]. Furthermore, an increase in TXNDC5 has been shown to down-regulate the serine threonine kinase, p38 MAPK. Likewise, a decrease in TXNDC5 levels causes an increase in MAPK stimulation, triggering adiponectin-induced phosphorylation in adipose tissue and synovial cells [49,50]. These findings indicate that the relationship between TXNDC5 and the adiponectin receptor 1 may be complex and variable according to tissue, and even within a given tissue, in terms of function, in order to achieve proper signal transduction of the adiponectin receptor.

### 3.4. Protein Interactions

TXNDC5, as a chaperone, interacts with a vast number of different proteins, having an important role in diverse cell processes. Although the methods used to discover these interactions (co-sedimentation, anti-tag coimmunoprecipitation, yeast-two-hybrid or enzymatic studies) do not enable us to unveil its biological role, this can be inferred by knowing the characteristics of proteins with which it interacts, as shown in Table 4. Thus, it may participate in histone modification, DNA transcription, mRNA splicing, cell cycle control, cell signaling, movement and transport, metabolism, and protein degradation. What is especially remarkable is its interaction with other chaperones, where TXNDC5 can act as a co-chaperone, or even take part in electron transfer, as explained above. Along the same lines, the role of TXNDC5 in protein degradation, interacting with ubiquitinated and neddylated proteins, should be pointed out [51,52,53].

As is outlined below, some of these interactions can play an important role in certain diseases such as diabetes, neurodegenerative illnesses, cancer, vitiligo or arthritis. For instance, dysregulation in TXNDC5 may affect the cell cycle, through the cycling CDK5, histones or transcription factors such as ATF2 or ZNHIT2, being a possible cause of cancer. Loss of TXNDC5 function could promote APP misfolding and, consequently, play a key role in neurodegenerative diseases; moreover, its interaction with the zinc finger XNF706 could be involved in schizophrenia. TXNDC5 interactions with NENF, PPP1R2, ALDOC, LDH, or PGD could explain its implication in diabetes [51,52,53].

**Figure 3:**
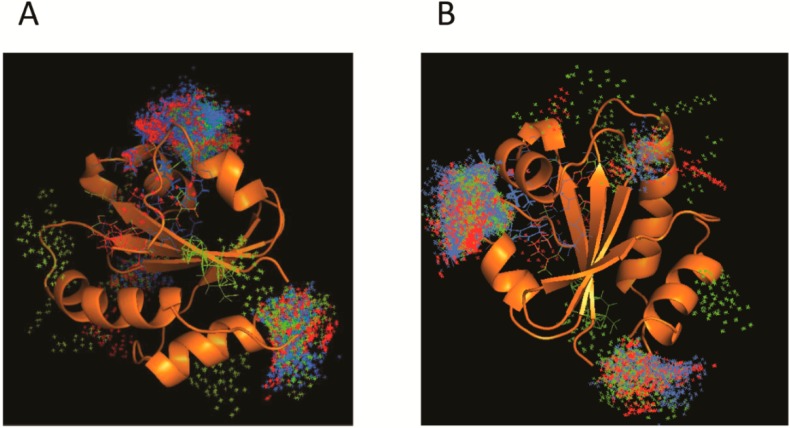
Current model of TXNDC5 interactions with different compounds. Panels (**A**) and (**B**) represent different angles of the docking assay using protein structure from Protein Data Bank [24]. First, many binding modes were generated by local and blind docking on the surface and in the cavities of the protein. Then, CHARMM (Chemistry at Harvard Macromolecules Mechanics) energies were estimated and most favorable energies were evaluated with FACTS (Fast Analytical Continuum Treatment of Solvation), clustered and visualized. Images show molecular coupling between TXNDC5 protein and the compounds cyclosporine A (blue), diazoside (red) and paracetamol (green). The interaction is mainly established within two domains of the protein involved in the action and its regulation [59,60].

Beside all the protein interactions, TXNDC5 can also be associated with multiple compounds that cause oxidative stress, some of which are shown in Table 5 [54]. In fact, a docking assay (Figure 3A,B) carried out with the third TXNDC5 catalytic domain as a target and three ligands (cyclosporine A, diazoxide and paracetamol) revealed that there are three main zones to establish the interaction. These zones might be a part of the active or regulatory site. Nevertheless, further folding, activity and stability studies would be required to verify this hypothesis.

**Table 4:** Protein interactions of TXNDC5 and cell processes involved.

Biological Process	Proteins
Histone deacetylation	HdAFX, HDAC10, HDAC2 and RPD
Transcription factors	ATF, BZW1,GNAI3, ZNF207, ZNF706 and ZNHIT2
Splicing	PRPF4
Cell cycle control	CDK5 and VCP
Cell signaling	NENF (MAPK1/ERK2, MAPK3/ERK1 and ATK1/ATK phosphorylase), PPP1R2 (PP1 regulatory subunit (inhibitor)), Adipo-R1, AdipoR2, YWHANG and YWHAQ
Cell transport	YecS (amino acid and cys ABC transport)
Cell movement	CSE1L, DBN1 and WF1
Ubiquitination	YOD1, HMG-20, CUL3, UBC, UBE2V1, UBXN1 and NPLOC4
Neddylation	DCUN1D1
Chaperones	HSP90AA1, TBCB, TGM2, TRMT1 and UNC45A
Carbohydrate metabolism	ALDOC, LDH and PGD
ATP	ATP6V1A
Amyloids	APP
Retrovirus	ENV

These interactions have been discovered by co-sedimentation, coimmunoprecipitation, enzymatic studies and yeast-two hybrid system [8,12,49,50,51,52,53].

**Table 5:** Interactions of TXNDC5 with different chemical compounds.

Type of Agents	Compounds
Carcinogens	2,3,7,8-Tetrachlorodibenzodioxine (herbicide), 4'-Diaminodiphenylmethane, Benzopyrene
Toxins	Aflatoxin B1
Immunosuppressor	Cyclosporine A
K^+^ channel activator	Diazoside
Adrenaline analogous	Isoprenaline
ROS	Nitric oxide
Oxocarbon	Trimellitic anhydride
PPARα	Pirinixic acid
Non-steroid anti-inflammatory	Paracetamol
Antibiotics	Quinolones
Anti-epilepsy drug	Valproic acid
Metals	Zinc
Vitamins	Ascorbic acid

Interactions among TXNDC5 and different organic and inorganic compounds, such as drugs and metabolites. All of them were experimentally discovered by different research groups [18,54,55,56].

## 4. TXNDC5 in Physiology and Pathology

### 4.1. Diabetes

Endocrine and exocrine pancreatic cells are known to have a high ER development, since this organelle is involved in folding, processing and exportation of enzymes and hormones. Several conditions, such as oxidative stress, can disrupt ER function causing accumulation of misfolded proteins [57]. As TXNDC5 helps in correct insulin folding, when its concentration decreases in pancreatic islets, the amount of insulin is reduced, causing diabetes, so TXNDC5 has a central role in glucose toxicity. Moreover, low TXNDC5 levels increase C/EBP-homologous protein (CHOP) and peIF2a concentrations, causing ER stress and consequently increasing misfolded proteins [9,57]. TXNDC5 also protects pancreatic cells from palmitic acid-induced apoptosis by decreasing ER stress response, so it has potential to become a new pharmacological anti-diabetes target [9].

In addition, there is some TXNDC5 dysregulation in macrosomic placenta, a fact associated with a high probability of developing type 2 diabetes [1,58].

### 4.2. TXNDC5 and Liver Diseases

Non-alcoholic fatty liver causes an accumulation of fatty acids in the liver (steatosis) independently of alcohol abuse. In some patients it can develop into other diseases such as steatohepatitis, cirrhosis and hepatocellular carcinoma [61]. TXNDC5, which has been associated with regulation of ER stress, is influenced by hepatic fat and might play an important role in apolipoprotein B (APOB) control and steatosis development [61]. Moreover the cellular lipid metabolism is crucial for hepatitis C virus entry, replication, assembly and release, as lipid droplets are involved in viral particle production and release. Consequently, TXNDC5 expression may also influence HCV replication by its role in lipid droplets [62].

Olive oil helps to reduce the accumulation of fatty acids thanks to the presence of squalene, an unsaponifiable lipid of this oil. It has been observed that high fatty acid levels, calcium deficiency and insulin resistance, may produce an ER homeostasis disruption, causing protein misfolding and generating oxidative stress. Squalene-supplemented diets in mouse models reduce fatty acid and cholesterol levels in liver while blood levels remain constant. In these models, it was also observed that the number and size of fatty droplets were both decreased. Moreover, TXNDC5 protein levels were found to be increased, while no changes in mRNA expression were detected. In conclusion, TXNDC5 and fatty acid levels are inversely related, even though this protein did not seem to contribute to reducing fatty droplet size. However, an increase in PDI-like chaperone expression, as with TXNDC5, may reduce misfolded protein and inclusion body accumulation [61].

### 4.3. Rheumatoid Arthritis

Some gene polymorphisms (96) were found to be correlated with a high rheumatoid arthritis (RA) risk. These SNPs include five coding SNPs, 4 SNPs in the 3'-UTR, 35 and 53 tag SNPs or SNPs in intron near the 5' end. Only 25 of the 96 SNPs are related to the development of RA or ankylosing spondylitis (another type of arthritis). A total of 9 SNPs (rs9505298, rs41302895, rs1225936, rs1225938, rs372578, rs443861, rs408014, rs9392189 and rs2743992) showed substantial association with RA, while 16 SNPs (rs1044104, rs1225937, rs1225938, rs372578, rs89715, rs378963, rs1225944, rs1225947, rs1238994, rs369086, rs408014, rs368074, rs1225954, rs1225955, rs13209404 and rs3812162) had a significant association with ankylosing spondylitis. Moreover, rs443861 polymorphism is significantly associated with the onset of RA. In addition, rs443861 is considered to be good a risk marker for the disease [10,11]. Most of these polymorphisms are situated in or near a non-coding region of *TXNDC5*.

TXNDC5 expression is increased in RA. Previous studies have found that TXNDC5 is increased under hypoxic conditions; so, in RA, a decrease in the oxygen supply causes TXNDC5 upregulation of expression, which can be observed in affected tissues, such as fibroblast-like cells, synovial fluids and blood [10].

As mentioned above, TXNDC5 may act as a cellular adapter interacting with Adipo-R1 and, in an indirect manner, with Adipo R2. Adiponectins are cytokine-like mediators produced in adipose tissue and synovial cells. They stimulate the release of chemokines, proinflammatory cytokines, prostaglandins, growth factors, and proteins of bone metabolism and of bone matrix remodeling. Therefore, activation of Adipo-R1 and AdipoR2 generates an increase in TNF-α, IL-1α, IL-1β, IL-17, which establishes a synergic relation in osteoclastogenesis and osteoclast function. This may explain the abnormal erosion found in RA [12,49,50]. Moreover, a rise in TXNDC5 levels causes a reduction in p38 mitogen-activated protein kinase phosphorylation. This fact contributes to inflammatory processes in response to TNF-α and IL-1 and plays a key role in lymphocyte migration to the injured tissues [49,50].

### 4.4. TXNDC5 and Cancer

Several *TXNDC5* polymorphisms have been associated with cancer. In this regard, two intronic SNP, rs1225944 and rs1225943, have been related to hepatocellular carcinoma in the recessive model and in the co-dominant 2, recessive and long-additive models, respectively, in the Korean male population [63]. They might affect mRNA splicing, generating a truncated protein. In addition, homologues of TXNDC5 isoform 1, such as hemofiltrate CC-Chemokine-2 (Hcc-2), have been found to be upregulated in hepatocellular carcinoma [3]. Likewise, rs9505298, rs7771314, rs2815128, rs13210097 and rs9392182 polymorphisms could be involved in cervical, esophageal and liver carcinomas, since they have been found in tumor tissues with a high TXNDC5 expression, a circumstance that could be linked to an increase in tumor growth and invasive potential [4]. Moreover, TXNDC5 seems also to be involved in the development of gastric cancer [5]. In varioliform gastritis TXNDC5 is upregulated at the gastric mucosa, while the mucosa remains healthy at basal levels. Not surprisingly, varioliform gastritis can evolve into gastric cancer [64]. Further evidence of this TXNDC5 tumorigenic action was obtained when researchers showed that its inhibition by shRNA administration reduced tumor volume and weight [8] and decreased growth and invasive potential [4], a finding that indicates that TXNDC5 could be used as a biomarker and therapeutic target for cancer treatment.

Tumor tissues are characterized by increased cell proliferation, angiogenesis and hypoxia. TXNDC5, as a protein induced by lack of oxygen, may be involved in these processes, acting as a tumor enhancer [4,64]. In fact, *TXNDC5* mRNA and protein expression has been found to be increased in cervical, uterine, colon, stomach, prostate, liver and lung cancer [5,7], and the protein was significantly upregulated in colorectal adenoma and cancer [6] as compared with TXNDC5 levels in normal mucosa. In these tumor processes, the interaction between the *N*-terminal region of TXNDC5 (amino acids 33 to 77) and Adipo-R1 (amino acids 1–70), which is induced by hypoxia, could play an important role as well. Specifically, in renal cell carcinoma, the ratio of Erp46/Adipo-R1 is increased in metastatic tissue compared with that from patients without metastasis [8]. Moreover, TXNDC5 expression is induced by ROS/RA stress and could be involved in pro-apoptotic pathways through the pro-oncogenic receptor NR4A1 in pancreatic tumor cells [65]. Likewise, TXNDC5 is required in the angiogenic function of endothelial cells. Erp46 interacts with TNF-α receptor 1 and controls TNF-α-induced endothelium angiogenic response. TNF-α enhances ERK1/2-mediated cell growth angiogenesis, raising AP-1 translation factor levels. TNF-α also controls cathepsin B expression and Ras activation. TXNDC5 reduces Ras^C118^, which limits Ras activation. Although these two observations seem to be contradictory, recent studies show that Ras^C118^-thiyl radical intermediate, rather than glutathionylated Ras, is the Ras species with the highest activity, and that glutathionylated Ras has an activity similar to that of native protein. Therefore, TXNDC5 could help to restore native Ras, allowing intermediate radical creation. Lowering TXNDC5 levels and levels of other PDIs would increase modified Ras accumulation, which could no longer be reduced and, consequently, could not be activated. Ras activation induces AP-1-dependent gene expression, contributing to endothelial cell angiogenesis in response to cytokines. Despite this fact, we can infer that an increase in TXNC5 expression may contribute to the angiogenic response in cancer [48]. In addition, TXNDC5 can act as an enhancer for pro-oncogene MYC expression. MYC/IGH (insulin-like growth factor gene 1) rearrangements and up-expression contributes to tumor autonomy in multiple myeloma [66].

Also related to the Ras pathway, TXNDC5 seems to be implicated in cell aging. Current research validates the view that blood components exhibit protein changes related to cell aging. The levels of stress proteins, such as TXNDC5, are decreased in mononuclear and endothelial cells of older donor blood, compared with blood of younger individuals. These changes regulate signal transduction of the downstream Ras pathway, inducin*g* Ras expression and increasing Ras protein levels, thus provoking Ras-induced senescence [67].

### 4.5. TXNDC5 and Neurodegenerative Diseases

Some *TXNDC5* polymorphisms (rs13873) and *BMP6-TXNDC5* polymorphisms (rs1225934, rs13873) are significantly related to schizophrenia due to a deficit in cluster 1, while cluster 2 is not modified [13].

*S*-nitrosylation is a nitric oxide-mediated, post-translational modification of cysteine residues with a physiological function in the cell. However, imbalance in *S*-nitrosylation rates reduces pro-survival proteins levels and is involved in neurodegenerative diseases. For instance, in neuroblastoma cells, TXNDC5 *S*-nitrosylation decreases, a circumstance that could modify multiple signal transduction pathways and compromise neuronal survival [68].

### 4.6. TXNDC5 and Vitiligo

Seven polymorphisms have been associated with non-segmental vitiligo (NSV). Four SNPs are intronic (rs1225943, rs1225944, rs1225945 and rs1225958) and three of them are found in the tenth exon (rs1043784, rs7764128 and rs8643). NSV is a polygenic disease thought to involve TXNDC5 [14]. Vitiligo is a skin disease characterized by a depigmentation produced by a progressive loss of melanocytes. The causes remain unknown, but it has been attributed to autoimmune reactions, neurodegenerative causes, toxicity, genetics or oxidative stress; though by far, oxidative stress is the most widely accepted. Reactive oxygen species (ROS) may be responsible for melanocyte damage, and are also one of the causes of protein misfolding. According to this hypothetical setting, stressed cells would not die and would continue to produce misfolded proteins, which would represent a possible cause of vitiligo [14].

## 5. Conclusions

The *TXNDC5* gene is located on the minus strand of *Homo sapiens* chromosome 6. The fact that it has been preserved in different species may be indicative of the relevance of its function. Several paralogous genes codifying for chaperones have also been identified. The *TXNDC5* gene codifies six transcripts by alternative splicing, but only two (*TXNDC5-001* and *TXNDC5-003*) encode protein. The two isoforms differ in the first 108 amino acids, present only in the first of them. This gene also encodes a conjoined gene called *MUTED-TXNDC5* that acts as a shRNA for *TXNDC5*.

The protein TXNDC5, belonging to the disulfide isomerase family, is a thioredoxin peroxidase mainly expressed in liver and endothelial cells, although it can also be found in many other tissues. Its main cellular location is ER, but, it can also be located in other organelles and even in the extracellular medium. It catalyzes the rearrangement of disulfide bonds through its Trx-like domain. These domains also participate in electron transfer between TXNDC5 and Ero-1-α or peroxiredoxin proteins, conferring an oxidoreductase capacity on this protein. On the other hand, TXNDC5 also has a chaperone activity independent of the Trx-like domain, contributing to the folding of secreted proteins in the membrane.

TXNDC5 may be related to several diseases. Some of its polymorphisms have been associated with the incidence of rheumatoid arthritis, vitiligo or neurodegenerative diseases. Furthermore, its modified expression in pancreatic cells may alter insulin folding and adiponectin response, which may be a new etiology for diabetes. It is also involved in colon, liver, lung and stomach cancers, due to its role in angiogenesis and cell proliferation under hypoxia conditions. Its upregulation may act as a tumor enhancer, while its downregulation reduces tumor morbidity. According to this panorama, this protein is expected to play an important role in pathology in future years.

## Supplementary Materials

Supplementary table can be found at http://www.mdpi.com/1422-0067/15/12/23501/s1.
